# The breaking point where repeat expansion triggers neuronal collapse in Huntington’s disease

**DOI:** 10.1016/j.xgen.2025.100816

**Published:** 2025-03-12

**Authors:** Michael D. Flower, Sarah J. Tabrizi

**Affiliations:** 1 Huntington’s Disease Centre and Department of Neurodegenerative Disease, UCL Queen Square Institute of Neurology and UK Dementia Research Institute, UCL, London, UK

## Abstract

Somatic CAG expansion drives neuronal loss in Huntington’s disease (HD), but how expansion results in pathogenesis has remained unclear. Handsaker et al.^1^ use single-cell RNA and repeat length sequencing to reveal a phased model of expansion and toxicity, highlighting a critical tipping point beyond 150 CAG repeats where neuronal identity collapses and cells die.

## Teaser

Somatic CAG expansion drives neuronal loss in Huntington’s disease (HD), but how expansion results in pathogenesis has remained unclear. Handsaker et al. use single-cell RNA and repeat length sequencing to reveal a phased model of expansion and toxicity, highlighting a critical tipping point beyond 150 CAG repeats where neuronal identity collapses and cells die.

## Main text

Huntington’s disease (HD) is the archetypal repeat expansion disorder, yet how somatic CAG expansions drive neurodegeneration has remained elusive. Handsaker et al.^1^ bring clarity with a single-cell study that measures CAG repeat lengths and transcriptomes in human postmortem striatum, providing an elegant explanation of HD progression through distinct phases of expansion and toxicity in medium spiny neurons (MSNs, or striatal projection neurons [SPNs] as the authors term them).

**Figure 1:**
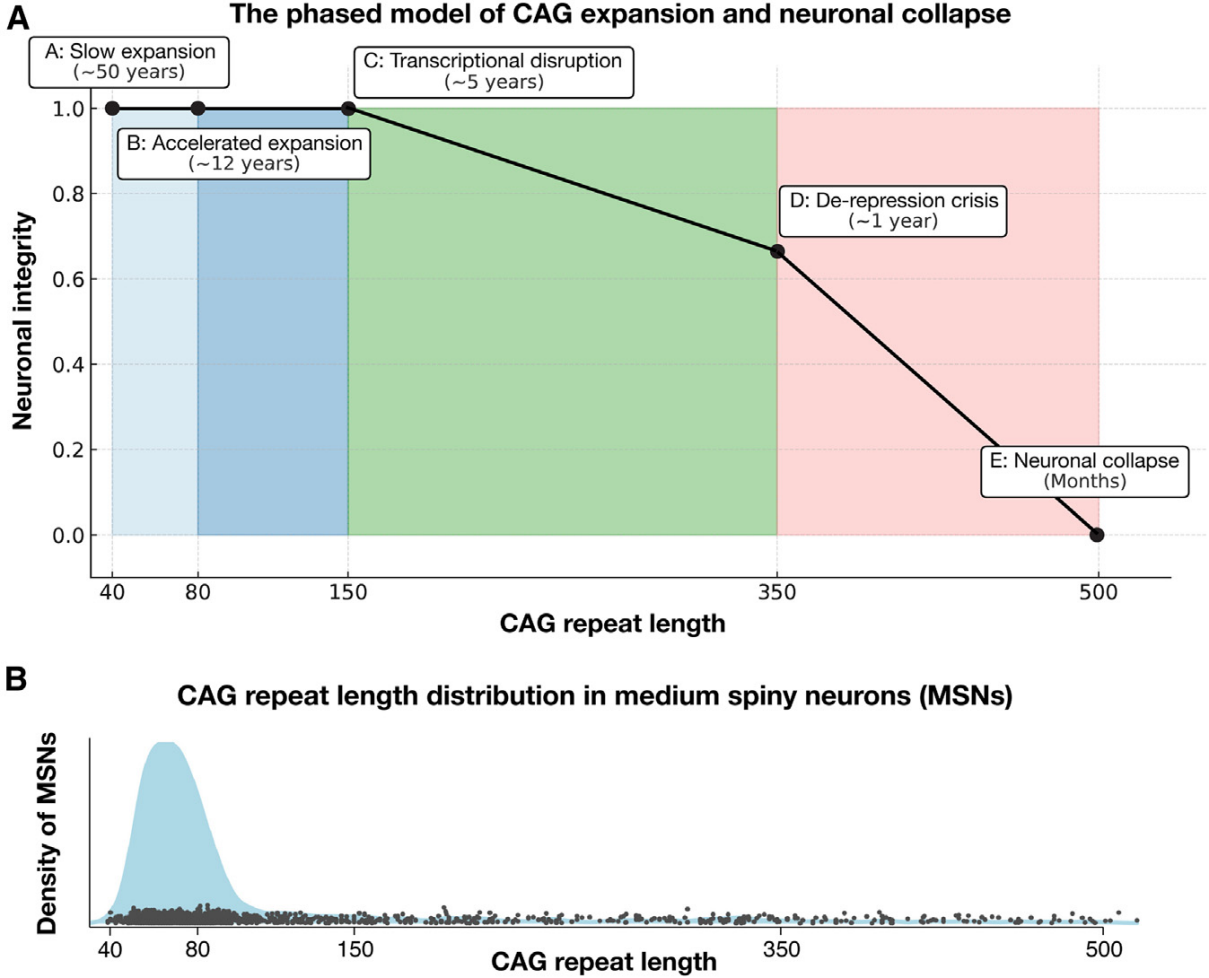
The phased model of CAG expansion and neuronal collapse in Huntington’s disease (A) Somatic expansion of the HTT CAG repeat progresses through distinct phases over a neuron’s lifetime. In phase A (40–80 CAGs), neurons remain stable with slow expansion. Phase B (80–150 CAGs) marks a rapid acceleration in expansion rate. Phase C (150+ CAGs) sees progressive transcriptional dysregulation, leading to the loss of neuronal identity. Phase D (350+ CAGs) is characterized by de-repression of embryonic and non-neuronal genes, triggering crisis. Phase E marks neuronal collapse and cell death. iSPNs are the most vulnerable to this process, which potentially explains their early degeneration in HD. This phased model provides a framework for understanding HD pathogenesis and therapeutic intervention. (B) The distribution of CAG repeat lengths across individual medium spiny neurons (MSNs, adapted from Handsaker et al.^1^) reflects the heterogeneity of somatic expansion at the time of death. This snapshot captures neurons at different points in the expansion process. MSNs spend over 96% of their life in phase A, where CAG expansion is glacially slow, before accelerating in phase B. A fraction of MSNs have exceeded the critical threshold (∼150 CAGs) and are in advanced stages of transcriptional collapse. However, this phase is rapid, and these neurons do not persist for long.

Their model resembles an ice sheet under relentless stress—stable for decades before fissures deepen and give way (Figure 1). Phase A is glacial, with expansion from 40 to 60 CAGs over 50 years—less than one CAG a year—akin to fine cracks forming in ice. By 80 CAGs, phase B sees those fractures widen as expansion accelerates 6-fold. Phase C marks the moment when the structure weakens irreversibly—neurons cross the 150 CAG threshold, and their transcriptional integrity starts to erode. Phase D is the collapse, as cracks turn to chasms, unleashing de-repression and senescence above 350 CAG repeats. Phase E swiftly follows—a terminal cascade where neurons, stripped of their defining features, perish.

Striatal neurons are healthy for more than 96% of their life—rapidly deteriorating only after crossing the 150 CAG threshold, leading to symptom onset (Figure 1). This phased model explains why small differences in inherited repeat length can produce large shifts in disease onset—longer repeats bypass slow expansion phases, racing toward collapse. Handsaker et al.^1^ also reveal that indirect pathway SPNs (iSPNs) suffer the greatest expansion and degeneration in HD. If the striatum is a vehicle, iSPNs are its brakes, controlling excessive movement. In HD, these brakes wear down first—perhaps explaining why early symptoms include chorea, a loss of motor restraint.

Mechanistically, expansion is thought to stem from DNA strand misalignment, forming slip-out structures that DNA repair complexes attempt to resolve through nicking, excision, and resynthesis. Imagine a zipper that has been pulled apart and then reconnected out of register; this creates small loops, or slip-outs, on both strands. To fix the misalignment, repair enzymes trim one strand, cutting out a section that includes one of the slip-outs, and use the remaining strand as a template to restore the sequence. When the slip-outs are close together like this, both can be removed in a single repair event, restoring the correct sequence. However, when CAG repeats exceed 70–90 units, the slip-outs form too far apart—beyond the typical range of excision—and are repaired separately. If the strand without a slip-out is removed, the looped strand serves as a template, incorporating extra bases and leading to an expansion. DNA repair enzymes show strand bias, favoring expansion over contraction, progressively worsening instability.^2^^,^^3^^,^^4^^,^^5^

This phased model shifts therapeutic priorities. It challenges conventional approaches, like lowering HTT protein; at any given time, most striatal neurons remain below the toxic threshold, with an HTT protein that supports cellular function, while those above perish within months. Instead, Handsaker suggests that HD pathogenesis is fundamentally a DNA-driven process, with neurons maintaining normal function throughout most of their lives as somatic expansion advances. This presents a wide therapeutic window, where even a modest slowing of expansion—even after symptom onset—could substantially slow progression.

However, questions remain. This study focuses solely on the striatum, leaving open whether repeat expansion follows the same rules elsewhere in the brain or in peripheral tissues. If toxic thresholds vary by tissue, repeat expansion might play a more or less central role in HD pathogenesis in other regions. The study captures only a single postmortem snapshot, meaning it cannot account for neurons that were already lost earlier in disease progression. Moreover, the model offers no answers on what specific toxic species emerges beyond 150 CAGs—whether it be toxic RNA, mutant HTT fragments, or aggregation-prone protein^6^—to dismantle the transcriptome and drive neuronal loss.

Handsaker’s findings also clash with fluorescence-activated nuclei sorting (FANS)-based postmortem studies, which found expansions in MSNs and corticostriatal interneurons but no correlation with cell loss, suggesting that expansion alone was insufficient.^7^^,^^8^ These studies used short-read sequencing, which is unable to detect very large CAG repeats, and pooled cells, effectively averaging out rare long expansions, masking their contributions. In contrast, Handsaker used long-read single-cell RNA sequencing with unique molecular identifiers (UMIs), which tags individual RNA molecules before amplification, correcting PCR bias that favors amplification of shorter repeats. This approach revealed rare catastrophically large expansions in MSNs, exposing a minority of neurons potentially driving disease progression.

Despite its gaps, Handsaker’s model reframes HD as a slow-burning fuse, where the greatest therapeutic gains may lie not in clearing the debris of the explosion but in slowing the fuse before it ignites.

## Acknowledgments

This work was supported by the CHDI Foundation (to M.D.F. and S.J.T.); the UK Dementia Research Institute (DRI), which receives its funding from UK DRI Ltd and is principally funded by the Medical Research Council, Alzheimer’s Society, and Alzheimer’s Research UK (to M.D.F. and S.J.T.); the UK Medical Research Council (MR/X008029/1 to S.J.T.); the Wellcome Trust (#223082/Z/21/Z to S.J.T.); and the Movement Disorders Foundation (to M.D.F.).

## Declaration of interests

The authors declare no competing interests.

## References

[1] Handsaker R.E., Kashin S., Reed N.M., Tan S., Lee W.S., McDonald T.M., Morris K., Kamitaki N., Mullally C.D., Morakabati N.R. (2025). Long somatic DNA-repeat expansion drives neurodegeneration in Huntington's disease. Cell.

[2] Genetic Modifiers of Huntington’s Disease (GeM-HD) Consortium (2019). CAG Repeat Not Polyglutamine Length Determines Timing of Huntington's Disease Onset. Cell.

[3] Usdin K., House N.C.M., Freudenreich C.H. (2015). Repeat instability during DNA repair: Insights from model systems. Crit. Rev. Biochem. Mol. Biol..

[4] Iyer R.R., Pluciennik A. (2021). DNA Mismatch Repair and its Role in Huntington's Disease. J. Huntingtons Dis..

[5] Wheeler V.C., Dion V. (2021). Modifiers of CAG/CTG Repeat Instability: Insights from Mammalian Models. J. Huntingtons Dis..

[6] Tabrizi S.J., Flower M.D., Ross C.A., Wild E.J. (2020). Huntington disease: new insights into molecular pathogenesis and therapeutic opportunities. Nat. Rev. Neurol..

[7] Matlik K., Baffuto M., Kus L., Deshmukh A.L., Davis D.A., Paul M.R., Carroll T.S., Caron M.C., Masson J.Y., Pearson C.E., Heintz N. (2024). Cell-type-specific CAG repeat expansions and toxicity of mutant Huntingtin in human striatum and cerebellum. Nat. Genet..

[8] Pressl C., Matlik K., Kus L., Darnell P., Luo J.D., Paul M.R., Weiss A.R., Liguore W., Carroll T.S., Davis D.A. (2024). Selective vulnerability of layer 5a corticostriatal neurons in Huntington's disease. Neuron.

